# Anti-aggressive effects of the selective high-efficacy ‘biased’ 5-HT_1A_ receptor agonists F15599 and F13714 in male WTG rats

**DOI:** 10.1007/s00213-015-4173-x

**Published:** 2015-12-23

**Authors:** Sietse F. de Boer, Adrian Newman-Tancredi

**Affiliations:** Department of Behavioral Physiology, Groningen Institute for Evolutionary Life Sciences, University of Groningen, P.O. Box 11103, 9700 CC Groningen, The Netherlands; Neurolixis Inc., Dana Point, CA USA

**Keywords:** Aggressive behaviour, Serotonin, 5-HT_1A_, Autoreceptors, Heteroreceptors, Dorsal Raphe, F15599, F13714

## Abstract

**Background:**

The serotonin (5-HT) deficiency hypothesis of aggression is being seriously challenged by pharmacological data showing robust anti-aggressive effects of 5-HT_1A_ receptor agonists in dose ranges that concomitantly inhibit 5-HT neurotransmission. Hence, an adequate interpretation of the role of 5-HT activity in regulating aggression depends on elucidating the predominant site of action, i.e., raphe presynaptic autoreceptors versus forebrain postsynaptic heteroreceptors, of these 5-HT_1A_ receptor agonists.

**Objectives:**

The present experiments investigated the anti-aggressive properties of the selective 5-HT_1A_ receptor agonists F15599 that preferentially target postsynaptic 5-HT_1A_ heteroreceptors in the frontal cortex and F13714 that more preferentially activates raphe somatodendritic 5-HT_1A_ autoreceptors.

**Methods:**

Both ‘biased’ agonists were acutely administered intraperitoneally in aggressive resident male WTG rats confronting an intruder.

**Results:**

Systemic administration of F15599 and F13714 exerted very potent (ID_50_ = 0.095 and 0.0059 mg/kg, respectively) anti-aggressive effects. At 4.5-fold higher dose ranges, the anti-aggressive effects were accompanied by concomitant motor inactivity and/or reduction of social engagement. Pretreatment with WAY-100635 counteracted the behavioural effects of both agonists.

**Conclusions:**

Overall, the qualitatively similar but quantitatively different anti-aggressive profiles of F15599 and F13714 largely correspond to their distinct 5-HT_1A_ receptor binding/activation potencies. Moreover, the marked anti-aggressive potency of F13714 adds additional support for a critical role of raphe somatodendritic 5-HT_1A_ autoreceptors, and hence phasic 5-HT neuron activity, in the initiation/execution of aggressive actions.

## Introduction

Ever since the initial discoveries that serotonin (5-hydroxytryptamine, 5-HT) serves as a neurotransmitter in the brain (Brodie et al. 1955) and that reduced levels of this indolamine (and/or its metabolite 5-hydroxyindole acetic acid, 5-HIAA) were found to be associated with aggressiveness in mice (Maas 1962; Valzelli and Garattini 1968). this evolutionary ancient and extremely well-conserved neurotransmitter system is generally considered the primary molecular orchestrator of aggressive behavioural traits in virtually every animal species, including man (see Duke et al. 2013 and de Boer et al. 2015 for review). However, the direction and exact causal linkage of this association is very complex, and it has proven notoriously difficult to unravel the precise role of this amine (and every facet of its synthetic and metabolic pathways, uptake and storage processes and dynamic receptor signaling mechanisms) in the predisposition for and execution of aggressive behaviour in both its normal and pathological forms.

For decades, high levels of aggressive behaviour were believed to be associated with low brain 5-HT neurotransmission activity (see Manuck et al. 2006; Coccaro et al. 2015 and Rosell and Siever 2015 for reviews). However, this seductively simple serotonin deficiency hypothesis of increased aggression is being challenged (de Boer and Koolhaas 2005; Carrillo et al. 2009; Duke et al. 2013; Takahashi and Miczek 2014; Takahashi et al. 2015) by a substantial database showing robust anti- or pro-aggressive effects of pharmacological agents in dose ranges that concomitantly inhibit or enhance, respectively, phasic 5-HT neuron firing and/or terminal release of 5-HT. In particular, agonists of 5-HT_1A_ subtype receptors are among the most potent drugs to suppress the initiation and execution of offensive aggressive behavioural displays in various vertebrate species ranging from fish, rodents, guinea pigs, canines to primates, including man (see de Boer and Koolhaas 2005 and Takahashi et al. 2012 for review and relevant Citations). This anti-aggressive effect only corroborates the 5-HT deficiency hypothesis of aggression under the assumption that these agonists would mainly act on the postsynaptic 5-HT_1A_ heteroreceptor population, i.e. mimicking higher 5-HT activity. However, the 5-HT_1A_ receptors are not only found post-synaptically as inhibitory heteroreceptor on various non-5-HT neurons in critical brain regions of the social behavioural (aggression) network, including the hypothalamus, amygdala, periaqueductal gray, septum and frontal cortex, but also as major inhibitory autoreceptor on the soma and dendrites of dorsal raphe 5-HT neurons (i.e. 5-HT_1A_ autoreceptor) that project to these forebrain areas. Hence, at the presynaptic somatodendritic level, these systemically applied agonists very effectively reduce 5-HT (re)activity, whereas at the level of the postsynaptic 5-HT_1A_ heteroreceptors they mimic the effects of an enhanced 5-HT signaling. This indicates that the autoregulatory negative feedback mechanism may obscure a simple linear relationship between aggression and 5-HT neurotransmission, and that a proper interpretation of the role of serotonin in regulating aggression depends on elucidating the predominant site of action of these 5-HT receptor agonists.

Furthermore, current drugs acting as full 5-HT_1A_ agonists are generally suboptimal in their specific anti-aggressive profile of activity because they indiscriminately activate 5-HT_1A_ receptors subpopulations in brain regions that mediate a host of other behavioural (locomotion, sleep, body care, appetite, sex), neuroendocrine (HPA-activation, prolactin release) and autonomic physiological responses (hypothermia, bradycardia and hypotension). Not surprisingly, therefore, the anti-aggressive effects of most full 5-HT_1A_ receptor agonists are usually accompanied with, or even may be primarily caused by, a concomitant reduction of initiative motor activity, social engagement and/or physiological thermogenic and cardiovascular functioning, e.g. rendering them behaviourally rather non-specific (de Boer et al. 2000).

However, some 5-HT_1A_ agonists can more selectively reduce aggressive behaviours without affecting other non-aggressive behaviours. For example, the highly selective but low efficacy 5-HT_1A_ receptor agonist S-15535, that generally acts in-vivo as a preferential agonist on 5-HT_1A_ autoreceptors but as a weak partial (ant)agonist on postsynaptic 5-HT_1A_ receptor sites (Carli et al. 1999; Millan et al. 1994). exerted a rather consistent and behavioural-selective reduction in aggressive behaviour over a wide dose range (Millan et al. 1997; de Boer et al. 2000). These data strongly indicate that the acute aggression-reducing effects of 5-HT_1A_ agonists are predominantly due to their action on the inhibitory autoreceptors, presumably attenuating a phasic social conflict (intruder)-activated 5-HT neurotransmission (de Boer and Koolhaas 2005; and discussion for further support of this view). Hence, it would be desirable to select compounds that preferentially interact with 5-HT_1A_ autoreceptor subpopulations mediating anti-aggressive properties, while minimizing interactions with 5-HT_1A_ heteroreceptor subpopulations that are predominantly involved in various other behavioural and/or physiological functions.

Recently, several exceptionally selective and high-affinity 5-HT_1A_ receptor agonists have been developed that are shown to very efficiently activate different subpopulations of 5-HT_1A_ receptors in the brain, presumably depending upon their coupling to G-protein subunits and/or intracellular second-messenger pathways, i.e. the so-called ‘biased’ or ‘functionally selective’ agonists (Newman-Tancredi 2011; Garcia-Garcia et al. 2014). For example, F15599 is an extremely selective and with high binding affinity (Ki = 3.4 nM) 5-HT_1A_ receptor agonist that exhibits preferential affinity for postsynaptic 5-HT_1A_ heteroreceptors in (prefrontal)cortical regions compared to presynaptic 5-HT_1A_ autoreceptors. On the other hand, its chemical congener F13714 has a similar high receptor selectivity and even 30-fold higher (Ki = 0.1 nM) receptor binding affinity than F15599 but seems to more preferentially activate the 5-HT_1A_ autoreceptor pool in the raphe nuclei (Newman-Tancredi 2011; Garcia-Garcia et al. 2014). Moreover, within cortical tissues, F15599 may even distinguish 5-HT_1A_ receptors expressed on glutaminergic pyramidal cells from those expressed on the GABAergic interneurons, an assertion based on the U-shaped dose–response curves seen after local microinjection of the drug into the medial prefrontal cortex in the forced swim test (Newman-Tancredi 2011) and resident-intruder aggression paradigm (Stein et al. 2013). Relevant for the current objectives is particularly the latter study demonstrating that local microinjection of low doses (0.03 and 0.2 μg) F15599 into the medial prefrontal cortex region of male mice were effective in reducing aggression but no effects were seen with higher doses (0.3 and 1.0 μg). This biphasic effect may be due to the fact that F15599 preferential activates 5-HT_1A_ receptors on GABAergic interneurons, causing a decrease of the inhibition of pyramidal neurons and thereby increasing activation of these principal medial prefrontal cortex projection neurons critically involved in the control of aggressive displays (Takahashi et al. 2015).

Regrettably, Stein and coworkers did not test F15599 after systemic administration or after local microinjection into the dorsal raphe nucleus, nor did they compare the F15599 effects with the divergently biased 5-HT_1A_ agonist F13714. Therefore, the objectives of this study were to assess and compare the possible anti-aggressive potencies of F15599 and F13714 in rats, both administered systemically and after microinjection into the dorsal raphe nucleus, with the main aim to further elucidate the relative contribution of somatodendritic autoreceptors and postsynaptic heteroreceptors in the potent anti-aggressive properties of 5-HT_1A_ receptor agonists. Special attention was paid to behavioural selectivity as well as receptor specificity of the anticipated anti-aggressive effects of these biased 5-HT_1A_ agonists by employing a detailed quantitative ethological analysis methodology and testing whether the biased agonists’ behavioural effects can be abrogated by the 5-HT_1A_ receptor antagonist WAY-100635. Finally, the behavioural profiles of F15599 and F13714 were compared with several previously scrutinized 5-HT_1A_ receptor agonist compounds in the WTG rat resident-intruder paradigm.

## Materials and methods

### Subjects and housing

Male wild-type Groningen (WTG) rats (*Rattus norvegicus*; originally wild-trapped animals and bred under conventionalized conditions for over 50 generations in our own facility) 4–5 months of age and weighing 379–475 g (average weight = 439 ± 5.0 g) were used as experimental subjects. This outbred strain is very suitable for social and agonistic behaviour studies because of their rich natural repertoire of social affiliative-aggressive behaviours that differ widely in intensity among individuals, ranging from no aggression at all to very high levels of aggressive behaviour (de Boer et al. 2003). Animals were housed in same-sex groups of 5–6 from weaning (23 days after birth) until the start (at age 145 days) of the experiments in clear type IV makrolon cages (60 × 60 × 20 cm). The cages were placed in a temperature-controlled room (21 ± 2 °C) with a fixed 12-h light/dark photoperiod (lights off at 10:00 h). All behavioural tests were performed in the dark phase (active period of rodents) between 11:00 and 13:00 h. The animals were allowed continuous free access to water and food. All procedures were conducted in conformity with the ethical rules of the Committee on Care and Use of Laboratory Animals (DEC) of Groningen University and European regulations (guideline 86/609/EEC).

### Experimental procedures

A resident-intruder agonistic paradigm was employed to induce offensive behaviour (see Koolhaas et al. 2013 for detailed procedure and video clips). Briefly, in this paradigm, male rats are housed individually in large observation cages (80 × 55 × 50 cm) with an oviduct-ligated (i.e. sterilized but hormonally intact) female to avoid social isolation and allow sexual behaviour, thereby facilitating territorial behaviour. After 1 week, the baseline level of offensive aggressive behaviour was tested on 3 consecutive days during maximally a 10-min confrontation with an unfamiliar male conspecific (WTG rats). The female partner of the experimental rat was removed from the home observation cage approximately 60 min prior to the start of this social provocation test. Naïve male WTG rats, socially housed in groups of 3 in transparent makrolon type IV cages, were used as conspecific intruder animals (average weight 372 ± 9.5 g and 3.5–4 months old). During the first 3 tests, the intruder was removed immediately after the first full attack from the resident and the attack latency time (ALT) was noted. Experimental groups were balanced on the basis of the ALT and the level of offensive behaviour performed during the fourth baseline test (day 4), during which the full range of behaviours was recorded and analysed (see below). Only animals that attacked (i.e. ALT <600 s) were included in the experimental groups. Non-attacking individuals (11 out of 144) were excluded from this study. On the next day (day 5), vehicle (sterile Ultra Pure water, *n* = 19 and *n* = 20) or either F15599 (0.0625, 0.125, 0.25, 0.5 and 1.0 mg/kg, IP, experiment 1, *n* = 45) or F13714 (0.003, 0.006, 0.012, 0.025, 0.062, 0.125, 0.250, IP, experiment 2, *n* = 50) was administered 30 min before the 10 min confrontation with a drug-free unfamiliar intruder conspecific and their behaviour was recorded again. In addition, in experiment 3, animals were tested 30 min after treatment with either vehicle/vehicle (UP), vehicle/F15599 (0.1 mg/kg) or F13714, WAY-100635 (0.3 mg/kg)/vehicle or WAY100635 (0.3 mg/kg)/ F15599 (0.1 mg/kg) or F13714. Each treatment group consisted of 6–8 subjects (*n* = 41 animals total).

During the resident-intruder agonistic confrontations on the non-drug test days 4 and on the drug test days 5, the full range of behaviours of the experimental resident was video-recorded and manually scored later on a custom-made data acquisition system (E-line) by an experimenter blind to treatment conditions. An extensive description of the different behavioural elements displayed during agonistic interactions has been reported previously (Koolhaas et al. 2013; de Boer et al. 2003). The frequencies and durations of the different behavioural elements were determined and expressed as a percentage of the total duration of the confrontation.

### Drugs

F15599 and F13714 compounds were provided by Pierre Fabre Pharmaceuticals, France (Lot # SBR1401003 and # JLM3001201). WAY-100635 was obtained from Sigma-RBI. All drugs were freshly (on the day of the experiment) dissolved in, and/or diluted with, vehicle solution (sterile and pyrogen-free UltraPure water (UP)) approximately 1.5 h before the start of the drug-testing experiments. The systemic injections are given intraperitoneally (IP) in a volume of 1 ml/kg body weight.

### Data analysis

Behavioural data were expressed as mean ± standard error (SEM). In the dose–response studies, the drug effects on each behavioural category were also computed as percentage of the respective vehicle-treated control values to enable comparisons between the various drugs. Regression analysis was used to calculate the dose that would elicit 50 % reduction in aggression (ED_50_ values). For most of the behavioural variables, a Shapiro-Wilkinson test for normality of the data indicated that the underlying population did deviate from a normal distribution. Hence, data were square-root transformed to normalize before parametric tests were performed. The dose-effect curves for each behavioural category of the offensive aggression test were analysed by a one-way ANOVA, with drug dose as between-subject factor. Further analyses were made by Dunnett’s test (comparison to the vehicle group) to determine the source of detected significance in the ANOVA’s. The criterion of significance was set at *P* < 0.05. All statistical analyses were performed using SPSS v.22 program.

## Results

### Offensive aggression test: general aspects in the undrugged state

Social confrontation initiated by the intrusion of an unfamiliar male rat into the home cage of the territorial experimental male counterpart resulted in the typical offensive aggressive and social behavioural repertoire displayed by the WTG resident rats: The resident male becomes aroused and quickly moves towards (approach) the intruder followed by social investigating, ano-genital sniffing and sometimes interspersed with bouts of grooming (particularly face-washing). Meanwhile, the intruder either actively explores the cage with rearing, sniffing and ambulatory movements, explores the resident or stays more passive/inactive by sitting still displaying only some scanning movements. Depending on the innate aggressiveness of the individual, the residents show piloerection and teeth-chattering and move towards the intruder while performing lateral/sideways threatening postures. In reaction to this, the intruder displays keep off movements and/or assumes an upright posture. In the latter case, the resident also adopts this posture maintaining piloerection and/or teeth chattering. These threatening displays are often followed by a clinch attack during which biting occurs towards the back, flank and neck regions of the intruder. These attacks usually lead to moving away (fleeing) of the intruder, which is then quickly followed (chasing) by the resident. Sooner or later, the intruder stops moving away and takes a submissive supine posture (signaling defeat) when approached and/or kept down by the resident. The resident male usually stops physically attacking when the intruder remains passive/supine. These fights generally occur in a bursting temporal pattern and intricately sequenced bouts of threatening/clinching/chasing are separated by periods of relative inactivity but alert scanning/observation. During these gaps, the resident engages in non-social behavioural acts such as walking, rearing, grooming or sitting still and scanning.

The latency time to the first attack in the undrugged residents (*n* = 175) on day 4 ranged from 12 to 600 s with a mean of 126 ± 28 s. In total, 14 of the 175 rats (8 %) did not initiate a clear attack and hence scored an ALT of 600 s (non-aggressive individuals). These animals were not further included in drug testing. During the 10-min test period, after the first clinch/attack-bite, undrugged resident rats spent 58 ± 4.3 % of the time on offensive aggressive behaviours and 15.5 ± 3.6 % on social explorative behaviours, thereby spending 73.5 ± 2.2 % on total social interaction. In the remaining part of the 10 min observation period, undrugged animals showed 16.5 ± 1.5 % non-social exploration, 6.7 ± 1.4 % inactivity and 3.2 ± 0.7 % grooming. The frequency of clinch attack bites in undrugged animals during the 10-min agonistic observation period amounted 18.3 ± 1.7.

### Dose–response effects of F15599

**Fig. 1:**
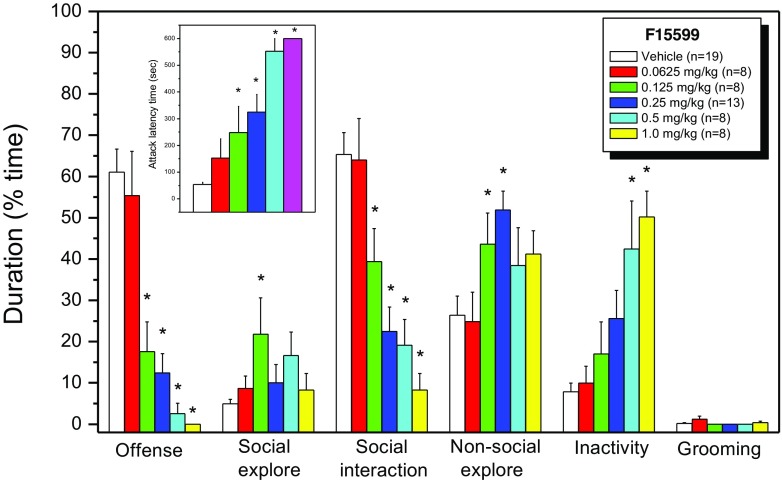
Effect of acute systemic (IP) F15599 on the attack latency time (ALT; *insert*) and different behavioural categories of resident wild-type Groningen rats in the offensive aggressive resident-intruder test. One-way ANOVA revealed significant effects of drug-dose for ALT [F(5,58) = 17.586; *p* < 0.0001], offensive behaviour [F(5,58) = 20.579; *p* < 0.0001], total social interaction [F(5,58) = 13.626; *p* < 0.0001], non-social exploration [F(5,58) = 3.381; *p* < 0.01] and inactivity [F(5,58) = 7.664; p < 0.0001]. *Asterisk* indicates that values are significantly (*p* < 0.05; Dunnett’s test) different from the vehicle value.

A similar behavioural pattern as in undrugged rats was observed on day 5 in the groups of rats injected with vehicle (UP). In contrast, treatment of the animals with 5 different doses of F15599 resulted in a significant and dose-dependent (MED = 0.125 mg/kg) delay in the latency to attack, and a potent reduction (ID_50_ = 0.095 mg/kg; Fig. 4) in the amount of aggressive behaviour directed towards the intruder rat (Fig. 1). This reduction in aggressive behaviour was, at 4.5-fold higher dose ranges, accompanied by a significant increase in behavioural inactivity scores (see Fig. 1) and only a slight (dose 0.125 mg/kg) compensatory increase in social exploration or non-social explorative behaviours (dose 0.125 and 0.25 mg/kg). Hence, a significant dose-dependent reduction in total social interaction was observed. Starting from the 0.25 mg/kg dose, F15599 induced clear signs of the so-called serotonin syndrome characterized by flat body posture, head-waving, lower lip retraction and hindlimb abduction, leading to increased behavioural inactivity scores and social disengagement (see Fig. 1). Basically, F15599-treated animals seemed to not take initiative for active behavioural exploration and/or actions anymore. Only upon physical provocation by the intruder animal, drug-treated residents started to display some active social explorative behaviours and in certain cases mild aggressive acts and postures.

### Dose–response effects of F13714

**Fig. 2:**
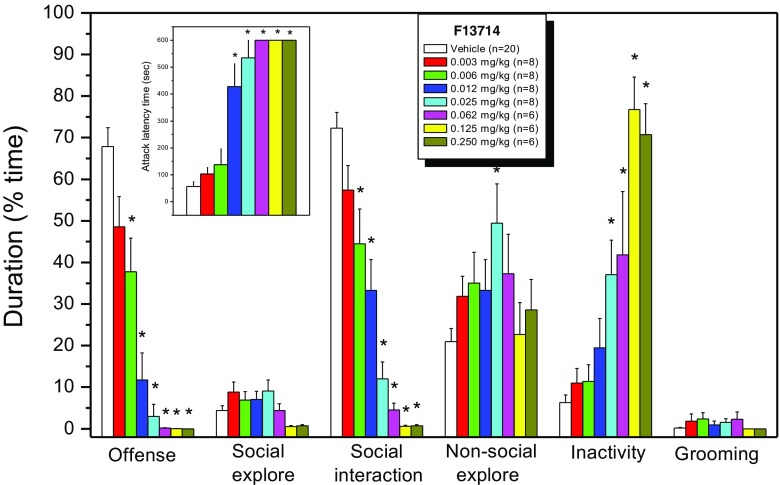
Effect of acute systemic (IP) F13714 on the attack latency time (ALT; *insert*) and different behavioural categories of resident wild-type Groningen rats in the offensive aggressive resident-intruder test. One-way ANOVA revealed significant effects of drug-dose for ALT [F(7,69) = 28.31; *p* < 0.0001], offensive behaviour [F(7,69) = 29.11; *p* < 0.0001], total social interaction [F(7,69) = 2.75; *p* < 0.002], non-social exploration [F(7,69) = 2.33; *p* < 0.01 and inactivity [F(7,69) = 17.86; *p* < 0.0001]. *Asterisk* indicates that values are significantly (p < 0.05; Dunnett’s test) different from the vehicle value

Treatment of the animals with 7 different doses of F13714 resulted in a significant dose-dependent delay (i.e. MED = 6 μg/kg) in the latency to attack and a pronounced suppression (i.e. ID_50_ = 0.0059 mg/kg; Fig. 4) in the amount (number and duration) of aggressive displays towards the conspecific intruder rats (Fig. 2). At slightly (4.5-fold) higher dosages, this anti-aggressive effect is accompanied by a potent increase (i.e. ED_50_ = 0.027) in behavioural inactivity (serotonin syndrome-like behavioural displays like flattened body posture, lower lip retraction and repetitive motor routines like head-waving and fore-paw treading) and unchanged social and non-social explorative behaviours; hence, total social interaction scores were concurrently significantly attenuated.

### Effective dose of F15599 and F13714 in combination with WAY-100635

**Fig. 3:**
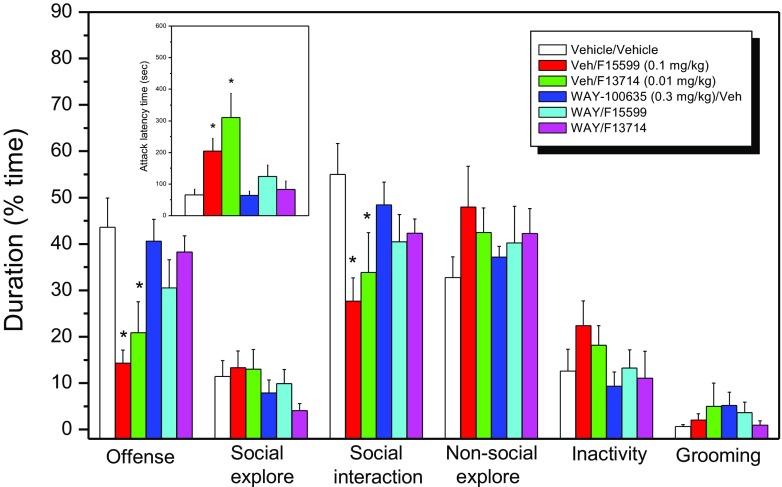
Antagonism of the anti-aggressive effects of F15599 and F13714 by pretreatment with WAY-100,635. One-way ANOVA revealed significant effects of treatment for ALT [F(5,34) = 6.32; *p* < 0.0001], offensive behaviour [F(5,34) = 4.36; *p* < 0.005] and total social interaction [F(5,34) = 2.70; *p* < 0.05]. Each treatment group consisted of 6–8 subjects. *Asterisk* indicates that values are significantly (*p* < 0.05; Dunnett’s test) different from the vehicle/vehicle values

Pretreatment with the selective 5-HT_1A_ receptor antagonist WAY-100635 (0.1 mg/kg) virtually completely prevented the anti-aggressive effects of the 0.1 mg/kg dose of F15599 and the 0.01 mg/kg dose of F13714 (see Fig. 3). While the behavioural performance of only WAY-100635-treated (0.1 mg/kg) animals was indistinguishable from both the vehicle-treated animals and their own undrugged behavioural profiles, treatment of animals with only F15599 (0.1 mg/kg) or F13714 (0.01 mg/kg) resulted in a significant delay of the latency to attack and a potent reduction in the amount and intensity of aggressive behaviours directed towards the intruder rat.

## Discussion

The present findings demonstrate that: (1) systemic administration of both F15599 and F13714 exerted very potent dose-dependent, but with considerable different potencies (ED_50_ = 0.095 and 0.0059 mg/kg i.p., respectively), anti-aggressive effects. (2) At slightly (4.5-fold) higher dosages, these anti-aggressive actions were accompanied by concurrent behavioural impairments, indicating only a modest separation from motor inactivity and reduction of social engagement. (3) The prototypical selective 5-HT_1A_ receptor antagonist WAY-100635 completely counteracted the behavioural effects of both agonists, demonstrating 5-HT_1A_ receptor mediation.

### Effects of 5-HT_1A_ biased agonists on aggressive behaviour

The present results add to the large database demonstrating that 5-HT_1A_ receptor agonists very efficaciously attenuate offensive-aggressive responses, even at low dose ranges that do not appear to cause motor impairments (de Boer and Koolhaas 2005). Furthermore, the qualitatively similar but quantitatively different behavioural profiles of F15599 and F13714 obtained in this study largely correspond to their previously reported distinct 5-HT_1A_ receptor binding and signaling potencies and partly support the ‘biased agonist’ profile of action reported for these compounds. Both F15599 and F13714 are extremely selective 5-HT_1A_ receptor full agonists with >1000-fold selectivity over other targets (Maurel et al. 2007) and with strikingly high in vitro binding affinities (Ki = 3.4 and 0.1 nM, respectively) (Newman-Tancredi et al. 2009). However, the 30-fold difference in binding affinity between F13714 and F15599 does not equally translate itself in in vitro receptor signaling activity or in various in vivo neurochemical, behavioural and physiological potencies. More specifically, in tests of signal transduction, F15599 activated Gαi-coupled receptors (EC_50_ = 110 nM) more potently and efficaciously than Gαo-coupled receptors (EC_50_ = 850 nM) while F13714 demonstrated a similar potency (although still roughly 150-fold and 1000-fold higher than F15599) for both Gαi (EC_50_ = 0.7 nM) and Gαo (EC_50_ = 0.8 nM) protein-coupled receptor activation (Newman-Tancredi et al. 2009). Subsequently, a large series of different in vivo studies have demonstrated that the in vitro signaling bias of F15599 to activate 5-HT_1A_ receptors translates to a preferential activation of cortical postsynaptic 5-HT_1A_ heteroreceptors with less influence on presynaptic 5-HT_1A_ autoreceptors. By contrast, F13714 exhibits an opposite preference, with more pronounced activation of 5-HT_1A_ autoreceptors and less potent activity at cortical heteroreceptors (Newman-Tancredi 2011; Garcia-Garcia et al. 2014). Consequently, this biased profile of action of F15599 will accentuate those behavioural and physiological effects that are assumed to be mediated by activation of postsynaptic heteroreceptors rather than presynaptic autoreceptors as summarized recently (Newman-Tancredi 2011). Thus, if activation of postsynaptic 5-HT_1A_ heteroreceptor populations were predominantly responsible for suppression of offensive aggression, F15599 should have exerted an anti-aggressive potency close to or even higher than F13714. However, our data clearly demonstrate a 16-fold *lower* anti-aggressive potency of F15599 compared to F13714 and this potency difference is roughly similar to or even higher than that observed for other behavioural and neurochemical effects (prepulse-inhibition deficit, 5CSRTT attentional deficit, inhibition 5-HT release) that are believed to be predominantly mediated by activation of raphe 5-HT_1A_ autoreceptors. Moreover, the anti-aggressive dosages of both F15599 and F13714 correspond to dose ranges that exert decreases in extracellular 5-HT in the hippocampus and prefrontal cortex with microdialysis studies, responses that undisputably are under rigorous 5-HT_1A_ autoreceptor control (Llado-Pelfort et al. 2010; Iderberg et al. 2015).

Interestingly, since it was shown that F13714 very rapidly (within just 3 days of treatment with 2.5 mg/kg/day) desensitized somatodendritic 5-HT_1A_ autoreceptors upon (sub)chronic administration Assié et al. (2006). it would be very relevant in future experiments to investigate the effectiveness of repeated and/or more sustained F13714 administration to suppress trait-like aggressiveness, particularly in subjects that demonstrate violent-like and excessive forms of aggression. Actually, one of the biggest problems of almost all behavioural pharmacological studies performed today is the predominantly acute treatment regimens, influencing the neurochemical target only for a very short period of time. If components of the serotonergic system are functionally and/or structurally adapted to (or basically underlying) the aggressive phenotype of an animal or human, it probably will take long-term treatment to reverse the activity to normal. On the other hand, (sub)chronic drug-induced alterations of the functional capacity of receptors, such as desensitization of 5-HT_1A_ autoreceptors, is a well-known phenomenon and may also lead to rapid or gradual tolerance to a drug’s proposed therapeutic efficiency.

### Separation of anti-aggressive and inactivity-inducing doses

**Fig. 4:**
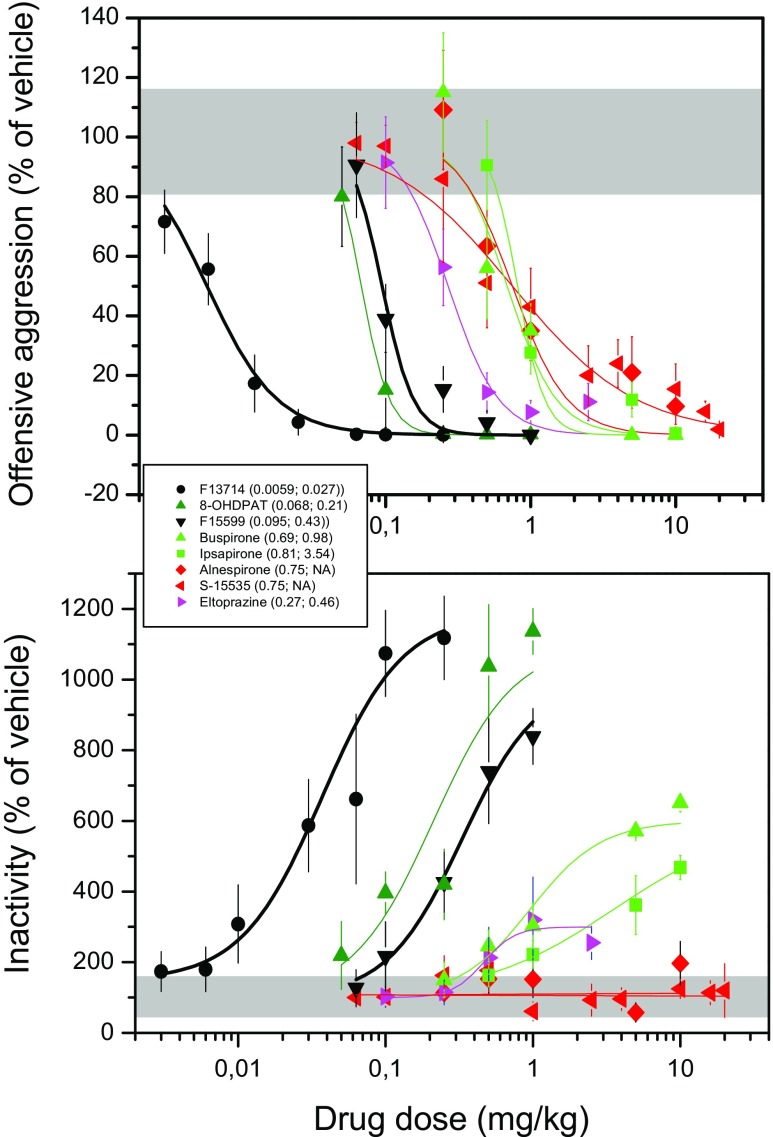
Comparative potency of different 5-HT_1A_ receptor agonists to inhibit offensive aggression (*top panel*) and to enhance motor inactivity (*bottom panel*) in the rat resident-intruder offensive aggression test. In the legend, the *numbers in between the parentheses* indicate the ED_50_ values of the drug’s anti-aggressive and inactivity-enhancing potency, respectively

Similar to almost all full 5-HT_1A_ receptor agonists tested so far in a wide variety of animals species and aggression paradigms, the present anti-aggressive actions of F15599 and F13714 at higher dosages are largely accompanied with slowed motor activity and reduced social engagement. Comparing the anti-aggressive ED_50_ values with the ED_50_ values of increasing behavioural inactivity scores (Fig. 4), it appears that all full agonists have a 4.5-fold separation between the anti-aggressive and pro-inactivity potencies. In general, it has been proven very difficult to clearly dissociate the anti-aggressive effects of full 5-HT_1A_ receptor agonists from their various other behavioural (locomotion, sleep, body-care, appetite, sex), neuroendocrine (HPA-activation, prolactin release) and autonomic physiological responses (hypothermia, bradycardia and hypotension). One reason for this very narrow ‘therapeutic’ window is likely due to the combination of the fact that most full agonists do not seem to sufficiently discriminate between different (i.e. pre-versus post- synaptic) subpopulations of 5-HT_1A_ receptors and their intrinsic high-efficacy to activate them. Hence, even the present highly efficacious but biased 5-HT_1A_ receptor agonists, F13714 and F15599, do not exert as favourable a serenic-like profile of action as has been observed for some other selective but low-efficacy 5-HT_1A_ agonists like S-15535 (de Boer et al. 2000). the mixed 5-HT_1A/B_ partial agonists like eltoprazine (Olivier 2004; de Boer et al. 2000) and more selective 5-HT_1B_ agonists like CP-94,253 (Miczek et al. 2004; de Boer and Koolhaas 2005). It should be noted, however, that the induction of typical serotonergic syndrome-like behaviours such as forepaw treading and flat body posture by F13714 and F15599 in WTG rats occurred at lower doses than those reported previously for these drugs in other rat strains (e.g. Sprague–Dawley, Assié et al. 2010). The dose separation between anti-aggressive and syndrome/inactivity-inducing effects could therefore vary in different rat strains. In addition, induction of serotonergic behaviours by 5-HT_1A_ agonists rapidly desensitizes upon repeated administration (e.g. Iderberg et al. 2015), emphasizing, once again, the importance of investigating the effects of such drugs under chronic administration regimens, as mentioned above.

### Importance of 5-HT_1A_ auto- and heteroreceptors in aggressive behaviour

Together with our previous work testing the anti-aggressive effects of several different 5-HT_1A_ receptor agonists (de Boer et al. 2000; De Boer and Koolhaas 2005). the marked anti-aggressive potency of F13714 in this study provides additional evidence that the anti-aggressive effects of systemic 5-HT_1A_ receptor agonists are largely expressed via their efficacious action on the inhibitory autoreceptors located on the soma and dendrites of raphe 5-HT neurons, presumably by attenuating intruder-activated 5-HT neuronal activity. Indeed, substantial evidence suggests that the actual display of offensive aggressive behaviour, or at least the intent thereof, is associated with (short-lasting) increased 5-HT neuronal activity. For example, robust increases of molecular neuronal activation markers in 5-HT-positive raphe neurons were found after the display of aggressive behaviour in rats and mice (Van der Vegt et al. 2003; Veening et al. 2005; Haller et al. 2005, 2006). Also, fMRI imaging in aggressively motivated rats has revealed a robust but shortlasting increase of the BOLD signal in the dorsal raphe area (Ferris et al. 2008). In addition, several in vivo microdialysis experiments in mice have clearly demonstrated that 5-HT release increased within the dorsal raphe nucleus, hippocampus and/or the medial prefrontal cortex when animals were aggressively aroused by social instigation and during escalated aggression after social instigation (Cadogan et al. 1994; Takahashi et al. 2011, 2015). Moreover, by employing wireless voltammetry techniques, phasic serotonin increases within the ventral striatum were found in resident rats confronting intruders (Nakazato 2013). Finally, direct pharmacological activation of 5-HT_1A_ autoreceptors in the dorsal raphe nucleus employing local microinjection of 5-HT_1A_ receptor agonists that potently suppress 5-HT neuron activity, consistently reduced aggressive behaviour in rats and mice, although in most cases also with a concurrent reduction in motor activity and social interactions (Mos et al. 1992; de Almeida and Lucion 1997; Van der Vegt et al. 2003; Calcagnoli et al. 2015). Preliminary findings employing direct intra- dorsal raphe microinjections of both F15599 and F13714 also revealed potent anti-aggressive effects (data not shown). This persuasive database support the view of rapid transient increases in 5-HT neuronal activity when an individual prepares or initiates imminent aggressive acts, and this view seems consistent with the classic theory on global brain 5-HT function that activation of the 5-HT system is most strongly related to general arousal and locomotor output (Jacobs et al. 2002; Beekman et al. 2005). However, the above view appears to be at odds with two other reports in the literature. One single and rather dated in vivo electrophysiological study in male tree shrews showed that serotonin neuron firing rates were reduced in dominant males immediately before an agonistic encounter (Walletschek and Raab 1982). Another very interesting recent pharmacogenetic study in male mice demonstrated that rapid suppression of 5-HT neuron firing by 5-HT_1A_ receptor agonist 8-OHDPAT treatment of genetically engineered mice expressing the 5-HT_1A_ receptor exclusively on 5-HT neurons led to a mild increase in aggressive responding (Audero et al. 2013). This particular data set cannot yet easily be reconciled with the above-outlined substantial database showing robust anti-aggressive effects of 5-HT_1A_ agonists in dose ranges that concomitantly inhibit phasic 5-HT neuron firing and terminal release of 5-HT. However, the recent rapidly advancing tools that enable measuring and manipulating neural activity of molecularly defined neuronal subtypes promise to elucidate the proposed causal relationship between dorsal raphe serotonin neuron activity and the various aspects of aggressive behaviour (Miczek et al. 2015).

Furthermore, it should be noted that aggression has to be conceptualized into two components, state-like aggression and trait-like aggressiveness. Whereas the latter refers to an individual’s personality and predisposition for acting aggressively, state-like aggression refers to the actual display of aggressive behaviours. While the normal initiation and display of offensive aggressive behaviour seems positively related to 5-HT impulse flow (i.e. state-like change), an inverse relationship probably exists between tonic 5-HT activity (i.e. trait-like phenomenon) and the likelihood to engage in impulsive and violent outbursts. Indeed, enduring lowered levels of 5-HT are generally reported to correlate with excessive levels and/or uncontrolled forms of aggressive conduct (see de Boer et al. 2015 for a further detailed review of this aspect). Based on the observations that high trait-like aggressive rats and mice are characterized by enhanced somatodendritic 5-HT_1A_ autoreceptor activity, a causal role was suggested for these autoreceptors in tonically suppressing brain 5-HT neurotransmission activity and the predisposition for high and excessive aggressive responding (de Boer et al., 2001; De Boer et al. 2009, 2015; Van der Vegt et al. 2001; Caramaschi et al. 2008). The recent molecular genetic studies by Gross and colleagues have indeed convincingly confirmed this causality by showing that adult transgenic mice with conditional overexpression of somatodendritic autoreceptors chronically suppressed 5-HT neural firing and phenotypically exhibited heightened aggressiveness (Audero et al. 2013).

Although the anti-aggressive effects of systemic 5-HT_1A_ receptor agonists are likely to be mostly mediated via the raphe 5-HT_1A_ autoreceptor activation, the activation of multiple populations of postsynaptic 5-HT_1A_ heteroreceptors by means of local microinjection into several key nodes of the aggression neural circuitry, including orbital and medial prefrontal cortex (Centenaro et al. 2008; Stein et al. 2013). hypothalamus (Cologer-Clifford et al. 1997), periaqueductal gray (Shaikh et al. 1997) and medial amygdala (de Almeida and Lucion 1997) have been shown to be effective in suppressing several forms of aggressive behaviour as well. However, activation of 5-HT_1A_ receptors in prefrontal cortical regions and septum (de Almeida and Lucion 1997) can also promote an increase in aggressive behaviour, suggesting the existence of functionally opposite post-synaptic 5-HT_1A_ heteroreceptor populations. Nevertheless, it is conceivable that after systemic administration of 5-HT_1A_ receptor agonists, activation of these postsynaptic heteroreceptors may contribute to the anti-aggressive effects as well. Actually, the postsynaptic 5-HT_1A_ heteroreceptor-mediated anti-aggressive effects may be accentuated in the situation when local 5-HT release is being attenuated due to the concomitant activation of the somatodendritic 5-HT_1A_ autoreceptors, and thereby also decreasing the activation of all other non-5-HT_1A_ receptors in the brain. In addition to local microinjection studies of 5-HT_1A_ receptor agonists in various brain regions to reveal the relative contributions of each population of 5-HT_1A_ receptor to the antiaggressive effects, another potential useful experimental design could be to systemically administer 5-HT1A receptor agonists and then locally block 5-HT1A receptors by microinfusion of the 5-HT1A receptor antagonist WAY-100635.

### Translational perspectives and Conclusions

Considering our emerging preclinical understanding of the psychopharmacology and neurobiology of aggression combined with the urgent clinical need to better and more efficaciously manage the rapidly increasing population of highly agitated and aggressive individuals with psychiatric problems and/or neurodegenerative disorders, it is quite disappointing to see relatively little clinical research effort and trials to bring selective 5-HT_1A/B_ receptor agonists into daily pharmacotherapeutic practice. Unfortunately, the current treatment of exaggerated aggression usually involves the prescription of combinations of drugs which by themselves are normally used to treat epilepsy (anticonvulsants), schizophrenia (anti-psychotics), anxiety (benzodiazepines) or depression (SSRI antidepressants) (Comai et al. 2012). Given the generally non-selective psychopharmacological profiles of these drug classes, it is not surprising that only poor therapeutic efficacy and/or a host of unwanted and adverse side effects have been reported for these drugs (Goedhard et al. 2006; Comai et al. 2012). Nevertheless, there are several reports that treating excessively aggressive and agitated patients afflicted with several neuropsychiatric disorders with rather non-selective 5-HT_1A_ receptor agonists (buspirone, eltoprazine) can be effective in reducing several measures of aggressive responding (Stanislav et al. 1994; Pfeffer et al. 1997; Ratey et al. 1991; Ricketts et al. 1994; Mak et al. 1995). Similarly, chronic treatment regimens with several different SSRIs that are all well known to indirectly lead to desensitization of 5-HT_1A_ autoreceptors were found to be variously effective in curbing aggressiveness and agitation in psychiatric and Alzheimer patients (Coccaro et al. 1997; McDougle et al. 1996; Vartiainen et al. 1995; Weintraub et al. 2015; Underwood and Fox 2014). Currently, there are no clinical studies available yet using selective and high-efficacy 5-HT_1A_ agonists as potential anti-aggressive agents. We therefore propose that (sub)chronic treatment with selective and highly efficacious ‘autoreceptor biased’ 5-HT_1A_ agonists like F13714 may have clinical potency to normalize (trait-like) excessive aggressive and violent conduct in patients with different psychiatric disorders or neurodegenerative diseases like Alzheimer’s.
